# Fire risk evaluation of heat-not-burn products and combustible cigarettes: Thermal exposure of different materials under ambient conditions

**DOI:** 10.1371/journal.pone.0351305

**Published:** 2026-08-07

**Authors:** Jauharah Khudzari, Sukport Sunan, Yee Guan Ng, Shahriman Abu Bakar, Zuradzman Mohamad Razlan, Rishan Murali, Muhammad Faiz Hilmi Rani, Shamsul Bahri Mohd Tamrin

**Affiliations:** 1 Centre of Excellence for Automotive and Motorsports, Faculty of Mechanical Engineering and Technology, Universiti Malaysia Perlis, Arau, Perlis, Malaysia; 2 Department of Environmental and Occupational Health, Faculty of Medicine and Health Sciences, Universiti Putra Malaysia, Serdang, Selangor, Malaysia; 3 Institute of Tropical Agriculture and Food Security (ITAFoS), Universiti Putra Malaysia, Serdang, Selangor, Malaysia; 4 Faculty of Mechanical Engineering, Universiti Teknologi Malaysia, Johor Bahru, Johor, Malaysia; University of California Davis, UNITED STATES OF AMERICA

## Abstract

Cigarettes have been reported to be a major cause of fire incidents and home fire deaths. Nowadays, alternative tobacco products such as heat-not-burn (HNB) are increasingly popular. In order to assess whether HNB products contribute to minimizing potential fire incidents, we evaluated the surface temperature profiles of HNB products during operation and cigarettes during smoldering or smoking, both when operated alone and when placed on different combustible materials. Experiments were conducted in Perlis, Malaysia, under ambient conditions (26.0°C ± 0. 8°C, 67% ± 4% relative humidity). The temperature of three HNB products and conventional cigarettes were recorded using a thermographic camera. Smoldering cigarettes reached a maximum temperature of 692°C and when smoked, the temperature increased by up to 22%. Puffing also led to an accelerated tobacco combustion in cigarettes. Simulated puffing had a minimal impact on the surface temperature of HNB devices, compared to that of cigarettes. The range of cigarettes temperatures observed in this study (617°C–844°C), were about 13 times higher than those for HNB products (47°C–65°C). Unlike cigarettes, HNB temperatures remained well below the reported ignition temperatures of all the combustible materials evaluated in this study. Under the conditions tested, the evaluated HNB products did not produce visible signs of thermal damage to the materials tested. Nevertheless, lit cigarettes caused visible thermal damage to fabrics, producing burnt marks on mats and dry matter indicating significant fire hazard potential.

## Introduction

According to a recent report by the National Fire Protection Association [1], most home fires and fire casualties in the United States resulted from one of five causes: cooking, heating equipment, electrical distribution and lighting equipment, intentional fire setting, and smoking materials. For the past few decades until 2020, smoking materials were clearly the leading cause of home fire deaths [1] and falling asleep while smoking was identified to be a primary human factor contributing to the deaths [2]. It was also reported that upholstered furniture and mattresses or bedding were among the first items to ignite fire, which is consistent with the primary areas of origin associated with fire deaths: living rooms and bedrooms.

Ignition temperature of a material plays an important role in determining the fire risk, with some materials igniting at lower temperatures than others. Cotton, for example, is one of the highly flammable fabrics [3] where the lowest temperature of ignition was at 215°C, yet widely available. Highly flammable materials can contribute to the rapid spread of fires. Furthermore, because cigarettes burn tobacco at around 800°C [4], improper disposal of, or accidental dropping of, burning cigarettes on flammable materials may ignite these flammable materials, resulting in devastating consequences.

**Fig 1:**
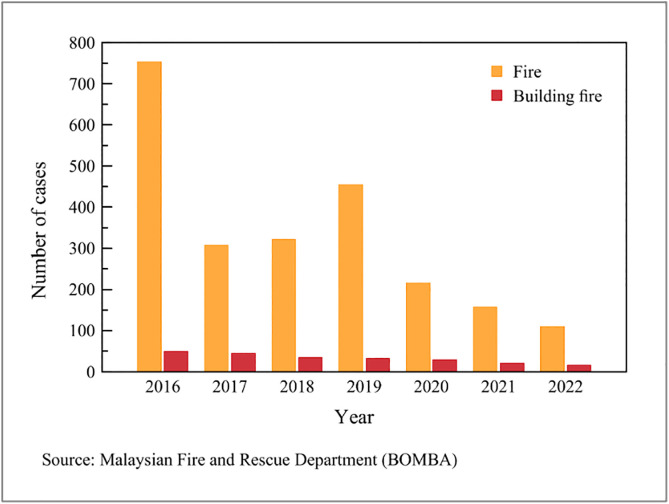
Number of fire cases caused by cigarettes between 2016 and 2022.

Environmental conditions can also impact the likelihood of initiating fires. Although Malaysia is known for its high humidity and abundant rainfall, certain regions (e.g., Perlis state) experience hotter and drier weather during the final phase of the Northeast Monsoon. These environmental conditions can increase the risk of fires initiating and spreading rapidly. The combination of high temperatures, low humidity, and strong winds can create perfect conditions for fires to ignite and quickly escalate and spread. In Malaysia, cigarettes ranked as the fourth leading cause of fires, as reported by the Malaysian Fire and Rescue Department [5,6]. The number of fire cases, however, has shown a declining trend since 2020 (Fig 1).

While combustible cigarettes are the most commonly available tobacco products, there is a growing trend in the market outlook for alternative products such as heat-not-burn (HNB) products that heat instead of combust tobacco. The market size of HNB products is projected to grow at a compound annual growth rate (CAGR) of 27.05% between 2022 and 2027 [7]. An HNB device is an electronic device designed to heat processed tobacco without combustion [8] via electronically controlled heating, which prevents combustion from occurring [9]. The HNB device operates at heating temperatures around 350°C [4] or below, which is significantly lower than the ignition temperature of tobacco which is 442°C [10].

The transition of consumers from traditional combustible cigarettes to HNB products may be attributed to several factors. These include the perception that HNB products are less harmful than combusted cigarettes and the belief that adopting HNB products can facilitate smoking cessation [11]. Additionally, users may find that HNB products are more socially acceptable due to its lack of ash, odor, and smoke [12].

With the rising demand for HNB products and the well-known fire hazards associated with cigarettes; it is crucial to conduct research to evaluate the potential fire risk of HNB products under Malaysian environmental conditions. To evaluate the fire risk, thermographic analysis can be used. Thermography applications have also been used in firefighting related studies [13], as well as in research related to cigarettes [14,15] and e-cigarettes [16,17].

**Fig 2:**
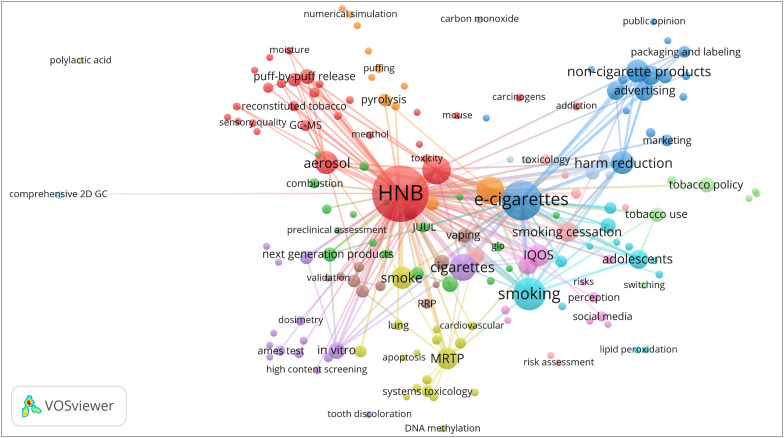
Popular topics in research articles related to heat-not-burn products. These topics (labels) were extracted from the author keywords of 895 research articles. The research articles were retrieved from the Scopus database on 20 February 2024 using an advanced search function. The primary search string was TITLE-ABS (“heat-not-burn” OR “heated tobacco” OR “tobacco heating” OR “tobacco vapour”). The minimum frequency of each label displayed on the VOSviewer map was set to three, resulting in a total of 166 keywords (research topics). The online map can be accessed at https://tinyurl.com/25rkkr6v.

**Fig 3:**
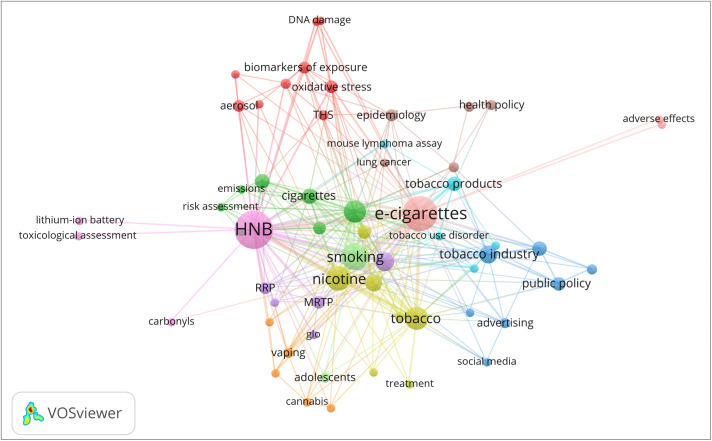
Popular topics in review articles related to heat-not-burn products. These topics (labels) were extracted from the author keywords of 146 review papers. The review papers were retrieved from Scopus database on 20 February 2024 by using an advanced search function. The minimum frequency of each label displayed on VOSviewer map was set to two, which resulted in a total of fifty-nine keywords (review topics). The online map can be accessed at https://tinyurl.com/2xpge4xd.

Prior to designing our research, a bibliometric analysis was conducted following the methods described in the literature [18]. Based on the bibliometric review on 1041 research and review articles indexed in Scopus database related to the used search criteria, previous studies have examined fire risks associated with combustible cigarettes and electronic cigarettes; however, direct experimental evidence on the surface-temperature behaviour and contact ignition potential of HNB products under tropical ambient conditions are according to our knowledge and research absent (Figs 2 and 3).

Studies and regulatory reports [19] on electronic cigarettes have highlighted fire and explosion hazards mainly associated with lithium-ion battery failure, overheating, charging faults, or misuse. The U.S. Fire Administration reported 195 e-cigarette fire and explosion incidents in the United States between 2009 and 2016, resulting in 133 acute injuries, while the U.S. FDA notes that vape fires and explosions, although uncommon, can be serious and may be battery related [20].

Other studies on e-cigarettes have examined heating-coil temperature and thermal degradation of e-liquids, including the formation of volatile carbonyl compounds at elevated coil temperatures. Talih et al. [17] reported heater coil temperatures ranging from 130°C to more than 350°C during direct-dripping e-cigarette use, while Geiss et al. [16] examined the relationship between e-cigarette heating-coil temperature and carbonyl emissions. While assessments of heating coil temperature may be implicitly linked to whether an electric device may be a fire hazard, they do not directly address the external surface-temperature profile, contact exposure, or ignition potential of HNB devices when placed on combustible household materials.

Although reviews of heated tobacco products have discussed product design, emissions, toxicant reduction claims, and health-risk assessment, fire-risk evaluation of HNB products remains comparatively underexplored [20]. Therefore, the present study contributes to this area by evaluating the potential fire risks linked to tobacco products under ambient conditions in Malaysia by analyzing the surface temperature profiles of HNB products during product operation and burning cigarettes during smoldering or smoking, both when operated alone and when placed on different combustible materials. The result of this study is expected to provide valuable scientific information to researchers, policymakers, and government agencies in Malaysia when considering potential regulations regarding HNB products and when educating the public about the risks associated with combusted cigarettes and the available alternatives to mitigate the identified fire risks. Additionally, this research will help fill a significant gap in the existing literature regarding the fire risk assessment of HNB products in tropical climates.

## Materials and methods

### Combustible materials

The selection of combustible materials in this study was made based on a diverse representation of commonly used materials in households. Five different fabrics were used, namely bamboo, cotton, linen, Tencel, and silk. These fabrics are 100% pure and chemical-free, certified by OEKO-TEX®. We also examined three mat materials, such as bamboo mat, PVC mat, and screw pine mat (*Pandanus atrocarpus*, also known as *mengkuang*) which are commonly available in Malaysia households.

These fabrics and mats were cut into 170 × 70 mm pieces and placed on top of sample holders. In addition, natural dry matters which consist of dry leaves and rice straw were also tested. Dry leaves were collected from Tecoma trees (*Tabebuia pentaphylla*) around the campus of Universiti Malaysia Perlis (Kampus Pauh Putra; GPS: 6°27’41.4“N, 100°21’16.1”E). Rice straw was collected from Fourstone Agro, Kedah (GPS: 6°19’50.1”N, 100°15’42.7”E). The moisture content (% wet basis) of dry leaves and rice straw was (3.00 ± 0.04) % and (2.11 ± 0.13) %, respectively.

### Heat-Not-Burn (HNB) products

In this study, we tested three HNB products and one conventional cigarette brand for comparison. Details on the tobacco products are shown in Table 1.

**Table 1:** Specification on the HNB and cigarette products used in this study.

Label	HNB device	Tobacco stick	Heating technology
HNB-OD	IQOS ORIGINALS DUO	HEETS	HEATCONTROL^TM^ technology Tobacco is heated by a ceramic blade inside the device.
HNB-LS	lil SOLID 2.0	Fiit	DUAL HEAT technology Tobacco is heated by a ceramic rod inside the device by induction heating.
HNB-IL	IQOS ILUMA	TEREA	SMARTCORE INDUCTION SYSTEM^TM^ The device is bladeless. A metal sheet is placed inside each tobacco stick and the tobacco is heated from within by induction heating.
Cigarette	–	Marlboro	Combustion

The HNB product referred to as HNB-OD (commercialized as IQOS ORIGINALS DUO with HEETS) uses controlled resistive heating technology, whereas the HNB products referred to as HNB-LS and HNB-IL (commercialized as lil SOLID 2.0 with Fiit and IQOS ILUMA with TERE*A*, respectively) utilize induction heating technology. HNB-IL is the only device with a bladeless design. The tobacco sticks designed for the HNB-IL cannot be used with any other device.

### Experimental design

**Fig 4:**
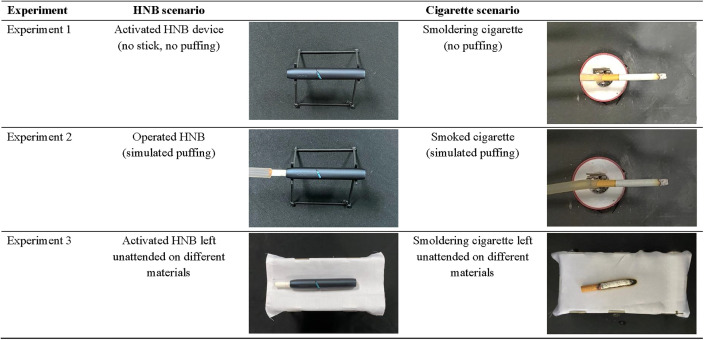
Multiple scenarios of HNB and cigarette setup in the experiments conducted.

Our experimental design comprised three sets of experiments considering multiple scenarios (Fig 4), specifically, HNB products in activation mode, HNB products in operation mode with simulated puffing, and activated HNB products left unattended on fabrics, mats, and dry matters. All experiments were conducted in triplicate, with 141 samples. The measured surface temperature profiles of the HNB products were compared to those of cigarettes.

Following common practice, the triplicate design was used to assess the repeatability of the thermographic measurements under controlled laboratory conditions. The uncertainty analysis showed that the relative variation of the recorded surface-temperature measurements was below 10%, indicating acceptable repeatability under the controlled experimental setup.

Data are presented descriptively as mean ± standard deviation (SD). No inferential statistical comparison was performed, as the purpose of the study was to characterize and compare surface-temperature profiles and visible material effects under defined experimental conditions, rather than to statistically test differences among materials or products.

### General experimental setup

**Fig 5:**
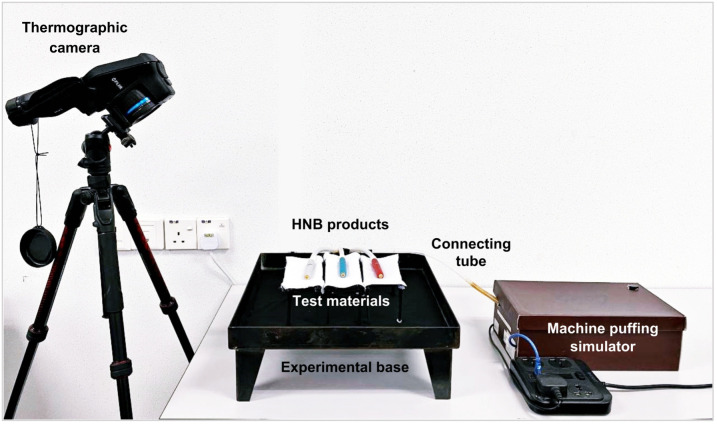
General experimental setup used for HNB products in Experiment 2. The same experimental setup is used in cigarette experiments.

Fig 5 shows the general setup of the experiments carried out in a laboratory located at Universiti Malaysia Perlis. Ambient temperature was measured to be around 26.0°C ± 0.8°C (min: 24.9°C and max: 28.5°C) and relative humidity at 67% ± 4%.

In Experiment 1, the HNB devices (HNB-OD and HNB-LS) were activated via the button on the devices without the sticks, and thermographic measurements were performed from activation to auto shut-off. However, Experiment 1 does not include HNB-IL as the switch-on function will only automatically activate when the associated stick is inserted inside the heating device. In Experiment 2, the HNB devices were operated with sticks connected to a machine puffing simulator. The machine puffing simulator was equipped with an Arduino microprocessor and a 12V DC air vacuum pump to simulate puffing.

The puff volume and puff duration were set at 55 mL and 2 second, respectively, following the Health Canada Intense (HCI) puffing regime [21]. However, the puff interval was modified from 30 s to 60 s which is the puff interval for the ISO 3308 puffing regime. The puff volume and duration were retained to represent an intense puff draw (with the atmospheric oxygen rich availability), while the interval was extended to provide a controlled and repeatable thermographic assessment of surface-temperature behaviour and to ensure that the consumption of a cigarette were comparable to the longest heating sequence of the HNB products tested (maximum 6 minutes). Thermographic measurements began as soon as the machine puffing simulator was turned on and ended when the HNB devices automatically turned off.

**Fig 6:**
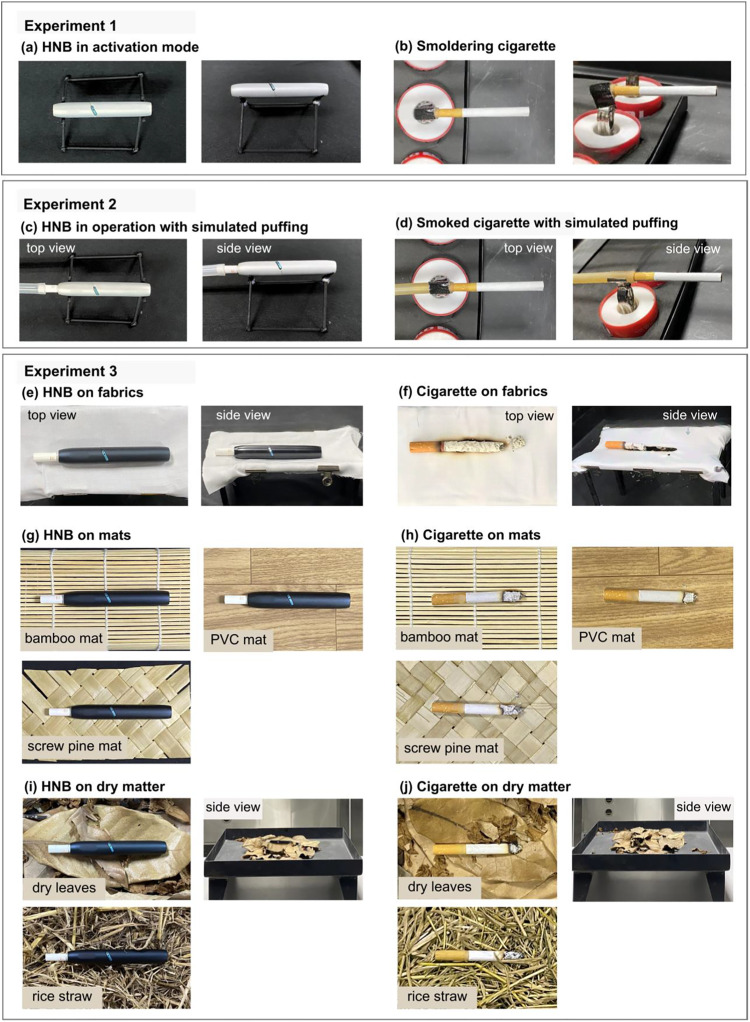
A close-up of HNB products. and cigarettes in each experiment. Experiment 1 (a, b), Experiment 2 (c, d), and Experiment 3 (e, f, g, h, i, j).

Experiment 3 involves operating HNB devices with sticks for three puffs. After that, the HNB were detached from the machine puffing simulator and being placed on ten different surfaces including fabrics (bamboo, cotton, linen, Tencel, silk), mats (bamboo, PVC, screw pine), and dry matter (dry leaves and rice straw). Sample holders were used in all experiments except for the application with dry leaves and rice straw. Close-up images of HNB products and cigarettes in each experiment are shown in Fig 6.

### Thermographic measurements

The surface temperature of HNBs and cigarettes were captured using a thermographic camera (E86, FLIR, USA). Prior to data collection, the camera calibration status was verified according to the manufacturer’s calibration record, and the camera was allowed to stabilize under laboratory conditions before measurement. A functional check was conducted before each experimental session by comparing the thermographic reading of a reference surface at ambient temperature with the value obtained from a calibrated thermometer.

To ensure high-precision thermal data, emissivity values were not generalized; rather, they were determined experimentally for each tested device casing and tobacco consumable using the reference tape method. A reference material (matte black tape) with a known baseline emissivity of 0.95 was affixed to the target surfaces. As the test objects were heated, the camera’s internal emissivity setting was manually adjusted until the thermographic temperature reading of the bare, un-taped material matched the true surface temperature recorded from the reference tape.

Through this calibration, specific emissivity settings were established and applied (refer to Table 2) to account for their distinct radiative properties. The reflected apparent temperature was set to match the measured ambient laboratory temperature. The measurement distance between the thermographic camera and the sample was maintained at approximately 0.5m, and images were captured with the camera positioned as perpendicular as possible to the measured surface to minimize angular measurement error.

**Table 2:** Emissivity settings for each device and their corresponding sticks.

Devices (Sticks)	Emissivity	
	(Devices)	(Sticks)
HNB-OD (HEETS)	0.91	0.94
HNB-LS (Fiit)	0.95	0.91
HNB-IL (TEREA)	0.94	0.88
Cigarette – Marlboro	0.88	

**Fig 7:**
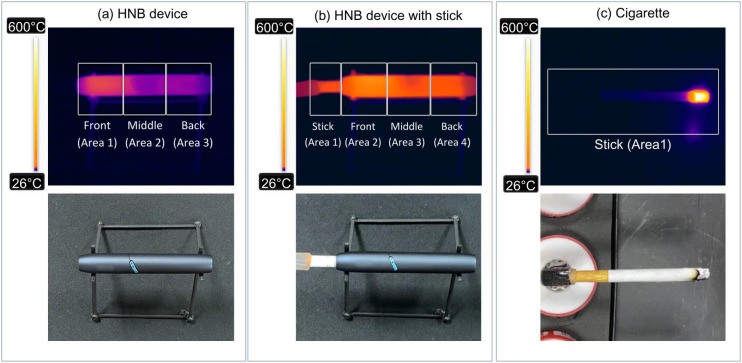
The position of analyzed thermal areas for (a) HNB device, (b) HNB device with stick, and (c) cigarette. The temperature scale presented in the image is intended to aid visualization; it does not represent the temperature range specified in the camera settings during the experiments.

The thermographic camera settings were also adjusted accordingly; the temperature range for HNB applications was −10°C to 120°C, whereas for cigarettes it was 300°C to 1500°C. To study the thermal distribution of the HNB devices, the thermal image was divided into three distinct thermal areas, as illustrated in Fig 7(a), while the whole HNB stick protruding from the device as shown in Fig 7(b) as well as the cigarette provided in Fig 7(c) were treated as a single area. Images and videos from the thermographic camera were processed in FLIR Thermal Studio Suite (version 2.0.6). The thermographic data was further analyzed using MagicPlot software (version 3.0). The results of HNB products were analyzed using Levenberg–Marquardt nonlinear least squares curve fitting algorithm [22].

### Ethical approval

Ethical approval was not required as the study did not involve any experiments on humans or animals.

## Results and discussion

### Thermal distribution of HNB devices

**Fig 8:**
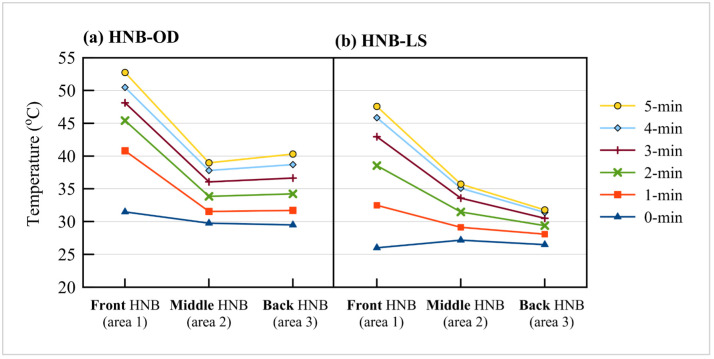
Surface temperature distribution of HNB devices during activation mode.

Fig 8 shows the temperature distribution of two HNB devices, HNB-OD and HNB-LS, during the initial five minutes of activation. The HNB-IL cannot be activated without inserting the stick. Clear differences can be observed between the two examined devices concerning both the magnitude and rate of surface temperature increase, implying distinct heating strategies and thermal control and management architecture. During the activation phase, the HNB-OD had a maximum recorded surface temperature of 52.7°C, which was 11% higher than for the HNB-LS (47.5°C).

Among the three analyzed areas of the HNB devices, the front part of the HNB showed the largest temperature difference (around 21.5°C) during device operation, implying the effect of heat transmission from the heating element located in that region. This effect was less pronounced at the middle and back ends of the devices. In addition, the temporal evolution of surface temperature differed between the two HNB devices examined. The HNB-OD device showed a faster increase in surface temperature than the HNB-LS device, rising by 9.3°C within the first minute of activation compared with 6.4°C for the HNB-LS device over the same interval. This difference may reflect variation in device design and heat-transfer characteristics, although the specific contribution of factors such as power input and thermal inertia was not directly assessed in this study.

### Comparison of surface temperature profiles of HNB products during activation and operation with simulated puffing

**Fig 9:**
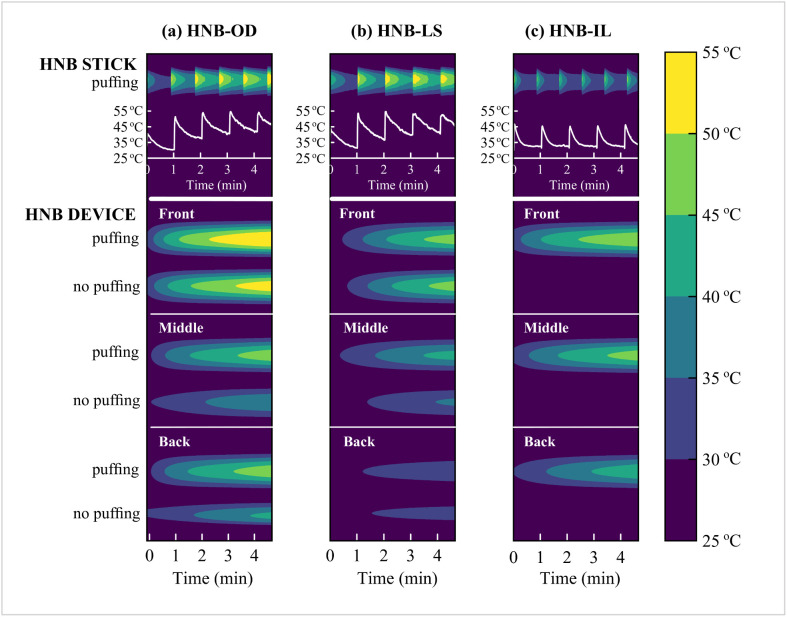
Temperature profiles of HNB products during activation mode (without puffing) and operation mode with simulated puffing.

Fig 9 illustrates how the components of HNB products (HNB sticks and HNB devices) were affected by simulated puffing. The highest surface temperature of the HNB sticks protruding from the device during operation was recorded for HNB-OD at 55.6°C, which was comparable to that for HNB-LS at 55.2°C. The recorded surface temperature of the HNB sticks had spikes following each puff; the temperature at the peak reached up to 69% of the temperature at the previous minimum point. HNB-OD showed the highest surface temperature changes of the HNB sticks with 18.8°C, followed by HNB-LS (16.5°C) and HNB-IL (14.1°C).

Similarly, due to the simulated puffing, the surface temperature of the HNB devices increased during operation with puffing compared to in the activation mode (without puffing). In both operation and activation modes, the front part of the HNB device experienced more heat compared to the middle and the back end. During operation, the highest surface temperature was recorded for the HNB-OD device at 57.0°C, followed by the HNB-IL and HNB-LS devices at 49.7°C and 47.6°C, respectively. Simulated puffing had a more noticeable effect on the surface temperature of the HNB-OD than HNB-OD which is indicated by the recorded temperature at the front of HNB-OD, showing an increase of 6.8% compared to only 0.2% in HNB-LS.

Overall, the results showed that HNB-OD consistently produced the highest measured surface temperatures compared to the other two HNB products. Our study has also identified that HNB-IL was the only product in which the surface temperature of HNB sticks and devices did not exceed 50°C under tested condition. These findings suggest that the design and construction of HNB-IL are more effective in concentrating the heat inside the device and stick, which can be explained by the induction heating technology with a bladeless design, as noted in Table 1.

The puffing regime used in this study was a hybrid/modified regime adapted from HCI (puff volume and duration at 55 mL and 2 second, respectively) and ISO 3308 machine puffing regimes (puff interval at 60 second. This adaptation was considered appropriate because the present study aimed to evaluate external thermal exposure and fire-risk potential during intermittent use and temporary placement of HNB devices, rather than to quantify mainstream smoke or aerosol constituent yields, for which strict adherence to the HCI regime would be more critical.

The 60 s puff interval was selected to harmonize the thermographic observation period between cigarettes and HNB products, as a strict 30 s HCI interval would accelerate cigarette consumption and cause the cigarette to reach the filter before completion of the HNB operating cycle. The influence of extending the puff interval from 30 s to 60 s was not directly quantified because a parallel strict-HCI comparison was not included. Nevertheless, this modification was necessary to ensure comparability of the thermographic observation period across product types (e.g.,: HNB products and cigarettes).

Under the modified protocol, puffing had limited influence on HNB surface temperatures, whereas cigarettes showed a marked temperature increase during puffing. Thus, while the modified interval should be acknowledged when comparing the findings with strict HCI studies, it is unlikely to affect the primary interpretation that the tested HNB products showed limited fire-risk potential under the evaluated conditions.

### Comparison of cigarette temperature profiles during smoldering and smoking

**Fig 10:**
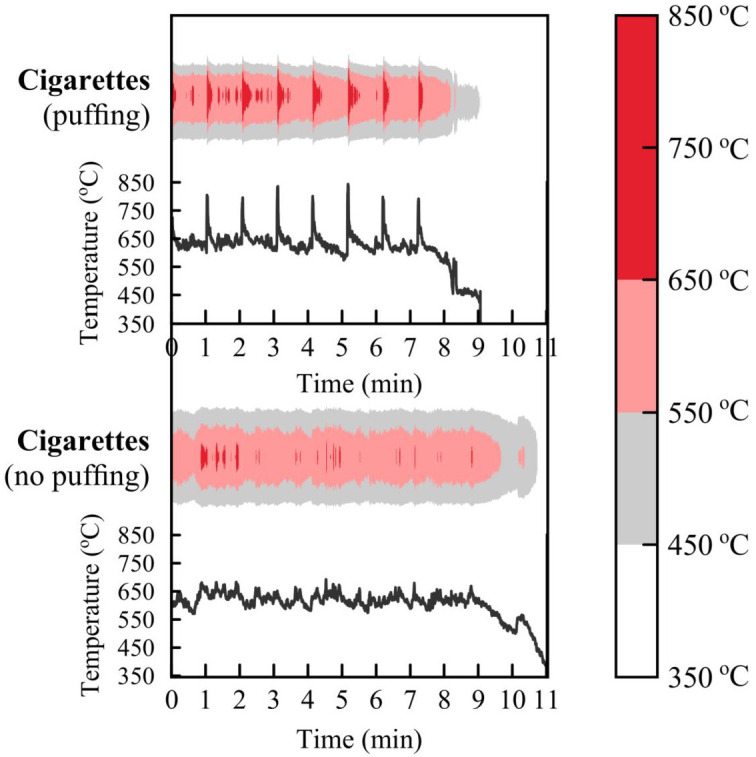
Temperature profiles of cigarettes with and without simulated puffing.

The average temperatures of cigarettes during smoldering (no puffing) and smoking (puffing) were 622.0°C ± 20.9°C and 637°C ± 20.9°C, respectively. The application of simulated puffing increased the surface temperatures of the cigarettes by 22%, indicating intensified combustion under forced-air flow intake; the temperature fluctuations during smoking were more noticeable, with higher peaks compared to those during smoldering, as shown in Fig 10. The results showed that when cigarettes were puffed (smoked cigarettes), the temperature reached up to 844.1°C, compared to 692.4°C without puffing (smoldering cigarettes). These elevated peak temperatures can be attributed to the increased oxygen intake and enhanced convection heat transfer due to puffing, which intensify exothermic kinetic pathways for combustion within the burning zone, promoting more vigorous combustion.

It was also found that under puffing conditions, the cigarettes were consumed approximately two minutes faster that during smoldering, due to the accelerated combustion of tobacco during puffing as evidenced by an earlier drop in the temperature down to 350°C. Together, these observations demonstrate that simulated puffing not only elevates combustion temperatures but also shortens the overall combustion duration by promoting faster tobacco consumption.

### Surface temperatures of HNB products and cigarettes during unattended operation on different combustible materials

Table 3 summarizes the average surface temperatures of HNB products and cigarettes during unattended operation when placed on various combustible household materials. Cigarettes showed the highest average temperature on silk and the lowest average temperature on PVC mats. The average temperatures of cigarettes when placed on the different materials ranged between 525.7°C and 599.9°C indicating sustained smoldering combustion under unattended operation.

**Table 3:** Average surface temperature of HNB products and cigarettes on different types of combustible materials. The values indicate the measured surface temperature of the front part of the HNB devices and cigarettes.

Materials	HNB-OD	HNB-LS	HNB-IL	Cigarette
	Average temperature ± SD (°C)			
Bamboo	59.9 ± 4.0	47.9 ± 1.9	45.8 ± 2.7	577.2 ± 21.4
Cotton	52.7 ± 3.4	45.6 ± 2.5	45.7 ± 2.5	591.8 ± 14.6
Linen	59.5 ± 4.0	46.7 ± 2.2	44.3 ± 2.6	599.8 ± 12.9
Tencel	54.4 ± 3.7	50.0 ± 2.8	45.6 ± 2.3	590.3 ± 24.3
Silk	59.4 ± 4.1	45.5 ± 2.4	43.7 ± 2.4	599.9 ± 20.6
Bamboo mat	53.7 ± 3.4	41.2 ± 1.5	46.8 ± 2.9	564.0 ± 43.9
PVC mat	53.7 ± 3.5	43.3 ± 2.5	44.5 ± 2.6	525.7 ± 45.1
Screw pine mat	54.8 ± 3.7	44.3 ± 2.6	44.4 ± 2.2	571.7 ± 30.2
Dry leaves	54.2 ± 3.7	42.8 ± 2.6	43.7 ± 2.7	545.3 ± 44.0
Rice straw	55.4 ± 4.1	45.3 ± 2.7	44.7 ± 3.0	596.5 ± 23.3

In contrast, all HNB products exhibited substantially lower surface temperatures with the recorded average temperatures below 60°C regardless of underlying materials. This pronounced temperature difference, exceeding an order of magnitude between cigarettes and HNBs explains the fundamentally different thermal regimes governing combustion-driven cigarettes versus electrically heated HNBs during unattended use. However, there was no clear pattern or systematic dependence in terms of which materials had the most influence on the recorded surface temperature because the results varied for each HNB product.

The highest average temperatures of HNB were observed on bamboo (59.9°C ± 4.0°C) for HNB-OD, Tencel (50.0°C ± 2.8°C) for HNB-LS, and bamboo mat (46.8°C ± 2.9°C) for HNB-IL. The range of surface temperatures is presented in Table 4.

**Table 4:** Range of surface temperature of HNB products and cigarettes on different combustible materials.

	HNB-OD	HNB-LS	HNB-IL	Cigarette
Material	Temperature range (°C)			
Bamboo	46.9–58.4	44.2–51.4	41.1–50.0	502.7–628.8
Cotton	47.0–59.3	40.8–49.1	39.7–47.4	544.1–623.3
Linen	47.7–60.2	42.1–49.9	39.9–48.4	563.4–636.9
Tencel	46.9–59.8	44.7–54.4	41.4–49.5	514.6–630.7
Silk	47.5–61.5	40.5–49.1	39.4–47.4	529.5–652.8
Bamboo mat	46.9–58.4	38.5–43.5	41.7–51.2	441.1–631.6
PVC mat	47.0–59.3	38.9–47.8	39.8–48.4	425.3–617.1
Screw pine mat	47.7–60.2	39.3–48.4	40.3–47.7	479.1–643.7
Dry leaves	46.9–59.8	37.7–46.5	38.8–48.0	453.2–634.8
Rice straw	47.5–61.5	40.3–49.8	39.2–49.5	523.8–642.9

**Fig 11:**
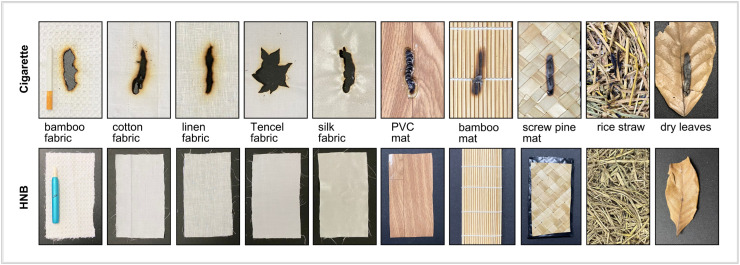
Material conditions after the experiments.

Additionally, Fig 11 made it clear that discarded burning cigarettes caused damage to fabrics and left obvious marks on mats and dry matter, indicating that significant thermal degradation or combustion of the material occurred. On the contrary, when HNB products placed on the combustible materials, no visible traces, discoloration marks or localized structural damage were observed on the surface of the materials, further confirming that the surface temperature of HNB products (during normal operation) remain insufficient to induce thermal degradation, smoldering or ignition of the tested combustible materials under the tested condition.

The difference observed on the combustible materials after having been exposed to burning cigarettes (damaged and significant burning marks) or HNB products (no marks) can be attributed to the corresponding high combustion temperature of cigarettes and the low operating temperature of HNB products compared to the ignition temperatures of the different combustible materials. Based on the available data in the literature (**Table 5**), the ignition temperatures of the tested combustible materials were all above 230°C, indicating that the low surface temperature of HNB products (below 65°C) is unlikely to cause any thermal damage to the tested materials and will not be able to ignite the combustible material.

**Table 5:** Ignition temperature of selected materials extracted from the literature.

Material	Ignition temperature (°C)
Cotton fabric	235°C (Tovarianskyi et al.) [3]
Rice straw	256°C (Zhaosheng et al.) [23]
Linen fabric	310°C (Zhou et al.) [24]
Bamboo fiber	363°C (Galaska et al.) [25]
Tencel fabric	450°C (Varga et al.) [26]
Silk fabric	600°C (Guan &Chen) [27]
Screw pine leaves	600°C (Diyana et al.) [28]
Dry leaves	800°C (Murray et al.) [29]
PVC mat	850°C (Font et al.) [30]

Furthermore, this also explains why cigarettes damaged all tested fabrics but had less effect (as seen in Fig 6) on other combustible materials (e.g., PVC). For example, ignition temperatures of fabrics (235°C – 600°C), are markedly lower than the ignition temperature of the PVC mat (850°C),. It was also noteworthy that all HNB devices are designed to have incorporated auto-shut off function by design, further reducing the likelihood of prolonged heating and rendering the operation inherently safer compared to cigarettes.

Based on the measured surface temperatures and the ignition temperatures reported for the tested combustible materials, the evaluated HNB products did not pose a fire hazard under the tested conditions. The very low surface temperatures of the assessed HNB devices which utilizes the electronic heating control make it reasonably likely that this observation may be generalized for other materials and common environmental conditions. However, it is important to highlight that this interpretation should be confined to the tested products, materials, and ambient conditions, and does not be apply to potential misuse scenarios, damaged devices, modified products, or extreme environmental conditions.

While the ignition temperatures of all tested combustible materials (>230°C) are significantly higher than the recorded surface temperatures of HNB products (<65°C), it is still of interest to also consider additional factors, such as material conditions and different environmental conditions, when evaluating the risk of initiating fires. For instance, old textiles may or may not be more susceptible to ignition than newer ones. Likewise, varying environmental conditions such as extreme heat and dry conditions may or may not also influence the heat transfer from HNB products to combustible materials. For the record, Malaysia experienced its highest temperature of 40.1°C in Chuping, Perlis, on April 9, 1998.

The present study was conducted under a defined range of ambient laboratory conditions in Perlis, Malaysia, with relatively stable temperature and relative humidity. Other environmental factors, such as wind speed, extreme ambient temperature, low relative humidity, and prolonged contact duration, may influence heat transfer between the tobacco product and the contacted material and hence the risk of lit cigarette but are unlikely to have substantial impact on the fire hazard of HNB devices.

While the findings from this study should be interpreted within the scope of the tested environmental conditions, products, materials, and exposure scenarios, it is reasonably likely that the results reported for HNB products in this study are also valid for most other materials and conditions given the low operating temperature and heating control of the tested products. To further contextualize and solidify the present findings for HNB products in general, additional efforts are needed recommended to examine other HNBs and potentially extreme scenarios conditions, as outlined above, which will be the subject of our future work.

For example, wind may alter convective heat loss from the product surface and may also affect smouldering or combustion behaviour, particularly for conventional cigarettes. Similarly, hotter and drier conditions may reduce the moisture content of combustible materials and potentially increase their susceptibility to thermal degradation or ignition. Prolonged contact with insulating, aged, dry, or degraded materials may also affect localised heat accumulation. These factors were not systematically varied in the present study and should be considered in future investigations.

Nevertheless, within the experimental conditions evaluated in this study, the recorded surface temperatures of the HNB products remained relatively low and substantially below the ignition temperatures of the tested combustible materials. Wind exposure may increase convective cooling at the external surface of HNB devices and further dissipate heat; however, this study did not systematically examine all possible environmental variations, including wind speed, extreme ambient temperature, low humidity, prolonged contact duration, and aged or highly dried materials.

It is worth noting that continued technological evolution and variability in real-world user behaviours are important factors to comprehensively assess the fire safety aspects of HNB products and operating conditions. The findings of this present study may help inform policymakers and regulators in developing evidence-based decisions and regulations related to the fire safety aspects of HNB products under typical usage scenarios.

## Conclusions

This study used thermography technique to evaluate the potential fire risk associated with cigarettes and HNB products under Malaysian environmental conditions. The surface temperature profiles of three HNB products were analyzed during activation, simulated puffing, and unattended contact with different combustible materials. The surface temperature profiles were also compared to those of conventional cigarettes and the ignition temperatures of common combustible materials. Among the tested HNB products, HNB-OD consistently exhibited the highest surface temperature in all experiments (maximum at 65°C). On the contrary, HNB-IL was the only model that did not exceed a surface temperature of 50°C under the tested conditions, suggesting the induction heating technology is effective at confining the heat within the device and stick compared to alternative product technologies tested in this study.

Across all HNB devices, the front end of the devices heated up more rapidly than the middle and back-ends section, consistent with closer proximity to the heating elements. Simulated puffing did not show significant effects on HNB temperatures due to the absence of combustion process. In contrast, cigarettes reached maximum smoldering temperature of 692.4°C which increased by 22% (844.1°C) during puffing. Overall, cigarettes had recorded temperatures between 617°C to 844°C, significantly (in order of magnitude) higher than those of HNB of 47°C to 65°C and substantially exceeded the ignition temperature of the tested combustible materials whereas the surface temperature of HNB devices remained far below the ignition temperatures of the tested combustible materials commonly encountered in household.

The findings indicate that, under normal operation and the ambient conditions evaluated in this study, the tested HNB products did not pose a fire hazard, unlike lit cigarettes which were found to be significant fire hazard. The surface temperatures of the tested HNB products remained substantially below ignition temperatures of the tested combustible materials during unattended operation where no visible thermal damage was observed following contact. Therefore, within the scope of the tested products, materials, and ambient conditions, HNB products are unlikely to pose a fire hazard associated with their surface temperature, whereas lit cigarette did.

Considering HNB products are still relatively new, numerous topics can be of interest. Future studies may include experiments with other HNB products, different temperatures, humidity levels, and wind speeds. Research may also be extended to investigate the effect of material conditions on flammability. Nevertheless, this study provides valuable scientific information to researchers, policymakers, and government agencies in Malaysia when considering potential regulations regarding HNB products, as well as when educating the public about the risks associated with combusted cigarettes and available alternatives to mitigate fire risk associated with tobacco products.

## Supporting information

S1 Table Surface temp dataset Exp 1. (XLSX)

S2 Table Surface temp dataset Exp 2. (XLSX)

S3 Table Surface temp dataset Exp 3 for Bamboo. (XLSX)

S4 Table Surface temp dataset Exp 3 for Cotton. (XLSX)

S5 Table Surface temp dataset Exp 3 for Linen. (XLSX)

S6 Table Surface temp dataset Exp 3 for Tencel. (XLSX)

S7 Table Surface temp dataset Exp 3 for Silk. (XLSX)

S8 Table Surface temp dataset Exp 3 for Bamboo Mat. (XLSX)

S9 Table Surface temp dataset Exp 3 for PVC Mat. (XLSX)

S10 Table Surface temp dataset Exp 3 for Mengkuang Mat. (XLSX)

S11 Table Surface temp dataset Exp 3 for dry leaves. (XLSX)

S12 Table Surface temp dataset Exp 3 for rice straw. (XLSX)

## References

[1] Hall S. Home structure fires. National Fire Protection Association; 2023.

[2] Ahrens M. Home fires started by smoking. Quincy, MA, USA: National Fire Protection Association; 2019.

[3] Tovarianskyi V, Adolf I, Petrovskyi V. Research temperatures of ignition and self-ignition of cotton and polyester fabrics. Fire Saf. 2021;38:32–7. doi: 10.32447/20786662.38.2021.05

[4] Paumgartten F. Heat-not-burn and electronic cigarettes: truths and untruths about harm reduction. Rev Assoc Med Bras (1992). 2018;64(2):104–5. doi: 10.1590/1806-9282.64.02.104 29641676

[5] KPKT. Time series statistics: MyKPKT 2017-2021. Strategic Planning and International Division, Ministry of Local Government Development. 2022.

[6] KPKT. Time series statistics: MyKPKT 2018-2022. Kuala Lumpur: Strategic Planning and International Division, Ministry of Local Government Development. 2023.

[7] Technavio. Heat-not-burn tobacco products market by product, distribution channel, and geography: Forecast and analysis 2023-2027. Infiniti Research Limited. 2023.

[8] Breathe. E-cigarettes, heat-not-burn and smokeless tobacco products 16. European Respiratory Society; 2020. 10.1183/20734735.ELF161 PMC7249783 32494306

[9] Smith MR, Clark B, Lüdicke F, Schaller J-P, Vanscheeuwijck P, Hoeng J, et al. Evaluation of the Tobacco Heating System 2.2. Part 1: Description of the system and the scientific assessment program. Regul Toxicol Pharmacol. 2016;81 Suppl 2:S17–26. doi: 10.1016/j.yrtph.2016.07.006 27450400

[10] Tibbitts TW. Ignition temperature of tobacco-a method of determination and relation to leaf burn. Tobacco Sci. 1962;6:170–3.

[11] Fung MDT, Diemert LM, Zhang B, O’Connor S, Schwartz R. Awareness and perceived risk of heated tobacco products. Tob Regul Sci. 2020;6(1):15–9. doi: 10.18001/trs.6.1.2

[12] Kim J, Lee S, Kimm H, Lee J-A, Lee C-M, Cho H-J. Heated tobacco product use and its relationship to quitting combustible cigarettes in Korean adults. PLoS One. 2021;16(5):e0251243. doi: 10.1371/journal.pone.0251243 33961641 PMC8104442

[13] Amon F, Pearson C. Thermal imaging in firefighting and thermography applications. Radiometric Temperature Measurements: II. Applications. 2009. p. 279–331.

[14] Gandhi S, Spivak SM, Pourdeyhimi B. Computer Aided Infrared Imagery for Fabric Surface Temperature Fields Under Simulated Cigarette Exposure. J Fire Protec Eng. 1995;7(4):107–23. doi: 10.1177/104239159500700401

[15] Yao ZY, Xu JC, Shen Y, Huang HQ. Effect of permeability of tipping paper on cigarette burning temperature and the property of mainstream smoke. Zhongguo Zaozhi Xuebao/Transactions of China Pulp and Paper. 2016;31:18–21.

[16] Geiss O, Bianchi I, Barrero-Moreno J. Correlation of volatile carbonyl yields emitted by e-cigarettes with the temperature of the heating coil and the perceived sensorial quality of the generated vapours. Int J Hyg Environ Health. 2016;219(3):268–77. doi: 10.1016/j.ijheh.2016.01.004 26847410

[17] Talih S, Balhas Z, Salman R, Karaoghlanian N, Shihadeh A. “Direct Dripping”: a high-temperature, high-formaldehyde emission electronic cigarette use method. Nicotine Tob Res. 2016;18(4):453–9. doi: 10.1093/ntr/ntv080 25863521 PMC6220833

[18] Md Khudzari J, Kurian J, Tartakovsky B, Raghavan GSV. Bibliometric analysis of global research trends on microbial fuel cells using Scopus database. Biochem Eng J. 2018;136:51–60. doi: 10.1016/j.bej.2018.05.002

[19] McKenna LA. Electronic cigarette fires and explosions in the United States, 2009–2016. U.S. Fire Administration, National Fire Data Center. 2017.

[20] Mallock N, Pieper E, Hutzler C, Henkler-Stephani F, Luch A. Heated tobacco products: a review of current knowledge and initial assessments. Front Public Health. 2019;7:287. doi: 10.3389/fpubh.2019.00287 31649912 PMC6795920

[21] Robinson RJ, Sarles SE, Jayasekera S, Al Olayan A, Difrancesco AG, Eddingsaas NC, et al. A Comparison between Cigarette Topography from a One-Week Natural Environment Study to FTC/ISO, Health Canada, and Massachusetts Department of Public Health Puff Profile Standards. Int J Environ Res Public Health. 2020;17(10):3444. doi: 10.3390/ijerph17103444 32429116 PMC7277227

[22] Murthy ZVP. Nonlinear regression: Levenberg-Marquardt method. In: Drioli E, Giorno L, editors. Encyclopedia of Membranes. Berlin, Heidelberg: Springer; 2014. doi: 10.1007/978-3-642-40872-4_1656-1

[23] Zhaosheng Y, Xiaoqian M, Ao L. Thermogravimetric analysis of rice and wheat straw catalytic combustion in air- and oxygen-enriched atmospheres. 2008.

[24] Zhou J, Zhu G, Gao Y, Gao S, Chai G. Experimental study on the effects of width and self-extinguishing on the upward flame spread for linen fabric. 2019.

[25] Galaska ML, Horrocks AR, Morgan AB. Flammability of natural plant and animal fibers: a heat release survey. Fire Mater. 2016;41(3):275–88. doi: 10.1002/fam.2386

[26] Varga K, Noisternig MF, Griesser UJ, Aljaz L, Koch T. Thermal and sorption study of flame-resistant fibers. Lenzinger Berichte. 2011;89:50–9.

[27] Guan J-P, Chen G-Q. Flame retardancy finish with an organophosphorus retardant on silk fabrics. Fire Mater. 2006;30(6):415–24. doi: 10.1002/fam.920

[28] Diyana ZN, Jumaidin R, Selamat MZ, Alamjuri RH, Md Yusof FA. Extraction and characterization of natural cellulosic fiber from Pandanus amaryllifolius Leaves. Polymers (Basel). 2021;13(23):4171. doi: 10.3390/polym13234171 34883674 PMC8659821

[29] Murray BR, Hardstaff LK, Phillips ML. Differences in leaf flammability, leaf traits and flammability-trait relationships between native and exotic plant species of dry sclerophyll forest. PLoS One. 2013;8(11):e79205. doi: 10.1371/journal.pone.0079205 24260169 PMC3832464

[30] Font R, Gálvez A, Moltó J, Fullana A, Aracil I. Formation of polychlorinated compounds in the combustion of PVC with iron nanoparticles. Chemosphere. 2010;78(2):152–9. doi: 10.1016/j.chemosphere.2009.09.064 19878966

