# Supplementary material for: Fire risk evaluation of heat-not-burn products and combustible cigarettes: Thermal exposure of different materials under ambient conditions
Source: PLoS One. 2026 Aug 7;21(8):e0351305. doi: 10.1371/journal.pone.0351305 (PMC13450766; doi:10.1371/journal.pone.0351305)
Supplement: Supplementary file 2 [file pone.0351305.s014.pdf]

UPM
UNIVERSITI PUTRA MALAYSIA
BERILMU BERBAKTI
FAKULTI PERUBATAN DAN SAINS KESIHATAN
FACULTY OF MEDICINE AND HEALTH SCIENCES

Date: 13th May 2026

Dr. Daniel Biftu Bekalo
Academic Editor
PLOS ONE

Dear Dr. Daniel,

Re: Revised submission of PONE-D-25-52903
Manuscript title: "Fire risk evaluation of heat-not-burn products and combustible cigarettes: Thermal exposure of different materials under ambient conditions"

Thank you for the opportunity to revise our manuscript for further consideration in PLOS ONE. We are grateful to the Academic Editor and the reviewers for their constructive comments and helpful recommendations. We have carefully revised the manuscript to address all editorial and reviewer comments.

In this revised submission, we have included the following files:

1. **Response to Reviewers**
2. **Revised Manuscript with Track Changes**
3. **Clean Revised Manuscript**

The manuscript has been revised to comply with PLOS ONE style requirements, including file naming and formatting. We have also reviewed and updated the funding information, financial disclosure, data availability statement, and supporting information presentation as required.

In response to the editorial requirements, we clarify the funding and role of funder statement as follows:

"The funders had no role in study design, data collection and analysis, decision to publish, or preparation of the manuscript. The funders provided vital information with regards to the HNB devices technology in order for the research team to conduct the experiments and derive the discussion."

Please find the Financial Disclosure Statement as follow:

i) The research grant was obtained by submitting a proposal to Philip Morris Malaysia.

ii) Upon approval, there were no specific grant award number being specified by the funder.

iii) The primary research team member who received the research grant was Ng Yee Guan as the project leader who manages the research fund under the Research Management Centre of the University.

iv) All research team members are students and/or employees of Universities as provided in their affiliation during the research project.

v) The website URL of the funder is as follow: https://www.pmi.com/markets/malaysia/en/about-us/overview

Following the integration of the Supplementary Information into the main manuscript, we have also revised the Data Availability Statement to state:

"All data are in the manuscript file."

Substantive revisions have been made in response to reviewer comments. These include clarification of the hybrid/modified puffing regime, specifically the use of the Health Canada Intense puff volume and duration with the ISO 3308 puff interval. We have also clarified the rationale for using a 60 s puff interval and acknowledged that the direct influence of this modification was not separately quantified because a parallel 30 s comparison was not included.

We have moderated categorical claims such as "no risk," "will never be able to ignite," and "practically absent" to ensure that the conclusions are appropriately bounded by the tested products, materials, and experimental conditions. The Discussion and Conclusion have also been expanded to address limitations related to the narrow range of environmental conditions, including the potential influence of wind, extreme temperature, humidity variation, prolonged contact duration, and material condition.

In addition, we have clarified the thermographic measurement parameters, including emissivity settings and calibration procedures. The statistical description has also been revised. The previous ANOVA statement was removed to avoid overinterpretation, and the study is now presented as a descriptive thermographic assessment supported by triplicate measurements and uncertainty/repeatability analysis.

We have also expanded the literature review to include related studies on combustible cigarettes, e-cigarette fire safety, and thermal behaviour of electronic nicotine delivery systems, while clarifying that the specific gap addressed by this study concerns HNB surface-temperature behaviour and contact fire-risk potential under tropical ambient conditions.

We believe that these revisions have strengthened the manuscript, improved methodological transparency, and ensured that the conclusions are appropriately supported by the data. We appreciate the time and effort of the Academic Editor and reviewers in evaluating our work.

Thank you for considering our revised manuscript.

Yours sincerely,

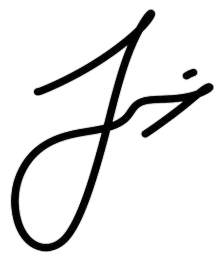

Associate Professor Dr. Yee Guan Ng (On behalf of all authors)
Department of Environmental and Occupational Health
Faculty of Medicine and Health Sciences
Universiti Putra Malaysia
43400 Serdang, Selangor, Malaysia
Email: shah86zam@upm.edu.my

RESPONSE TO REVIEWER

Academic Editor:

1. Please ensure that your manuscript meets PLOS ONE's style requirements, including those for file naming.	We've edited the manuscript in accordance with the PLOS ONE's style requirement including file naming.
2. We note that the grant information you provided in the 'Funding Information' and 'Financial Disclosure' sections do not match.	We've stated that this research work (of which this manuscript produced) was funded. We've corrected the funder as Philip Morris Malaysia instead of Philip Morris International as the grant was obtained locally from the funder in Malaysia.<br><br>However, there was no specific grant numbers available for the awards received. Hence, the award number in the submission system was stated as "Not Applicable"
3. Please state what role the funders took in the study. Please include this amended Role of Funder statement in your cover letter; we will change the online submission form on your behalf.	The funders had no role in study design, data collection and analysis, decision to publish, or preparation of the manuscript.<br><br>The funders provided vital information with regards to the HNB devices technology in order for the research team to conduct the experiments and derive the discussion.
4. Please provide a complete Data Availability Statement in the submission form, ensuring you include all necessary access information or a reason for why you are unable to make your data freely accessible.	"All data are in the manuscript file."<br><br>This statement has been added into "Data Availability Statement."
5. When completing the data availability statement of the submission form, you indicated that you will make your data available on acceptance. We strongly recommend all authors decide on a data sharing plan before acceptance, as	The data from Supplementary files has been integrated into the main manuscript file. All data has been made available within the manuscript.

the process can be lengthy and hold up publication timelines. Please note that, though access restrictions are acceptable now, your entire data will need to be made freely accessible if your manuscript is accepted for publication.	
6. Please include captions for your Supporting Information files at the end of your manuscript, and update any in-text citations to match accordingly.	The Supplementary Information file has been removed from submission as they now have been integrated into the main manuscript.
7. If the reviewer comments include a recommendation to cite specific previously published works, please review and evaluate these publications to determine whether they are relevant and should be cited. There is no requirement to cite these works unless the editor has indicated otherwise.	Not relevant as there were no specific previously published work recommended by reviewers to be included.

Reviewer 1:

The authors employed a modified version of the HCI method as the puffing regime.	Yes, we've now expanded this information specifically that the setup for the puffing in this experiment is a hybrid/modified puffing regime which utilize puffing volume (55mL) and duration (2s) from HCI while the puffing interval (60s) based on ISO 3308.<br><br>Refer to Line 186 – 192 (Clean Copy).
It would be helpful if the manuscript could briefly clarify the rationale for adjusting the puff interval from 30 seconds to 60 seconds...	This modification was made because the present study focused on thermographic assessment of external surface-temperature profiles and fire-risk potential, rather than mainstream smoke or aerosol constituent yields.

	The 60 s interval is also consistent with the interval used in the ISO machine-smoking regime and was selected to provide a controlled and repeatable thermographic testing condition.<br><br>In addition, if the strict HCI interval of 30 s was used, the combustible cigarette would be consumed more rapidly and could reach the filter before the HNB device completed its operating cycle.<br><br>Refer to Line 285 – 293 (Clean Copy).
...and indicate whether this modification had any measurable influence on the results.	The influence of this interval modification was not directly quantified in the present study because a parallel 30 s HCI comparison was not included. However, under the modified 60 s interval, simulated puffing produced only limited changes in the surface-temperature profiles of HNB products, whereas combustible cigarettes showed a clear increase in temperature during puffing compared with smoldering.<br><br>In the present study, cigarette temperature increased by up to 22% during simulated puffing, while HNB product surface temperatures remained substantially lower and below the ignition temperatures of the tested combustible materials.<br><br>Therefore, the modified puff interval should be considered when comparing the results with strict HCI emission studies, but it is unlikely to alter the main interpretation of limited fire-risk potential for the tested HNB products under the evaluated conditions.<br><br>Refer to Line 293 – 300 (Clean Copy).

<u>Reviewer 2:</u>

1. The study was fully funded by Philip Morris International, and the conclusions clearly indicate an advantage for HNB	The funders had no role in study design, data collection and analysis, decision to publish, or preparation of the manuscript.

products. Please clearly state the sponsor's role in study design, data collection, analysis, and manuscript preparation. This is crucial to maintaining transparency and demonstrating the absence of any conflict of interest.	The research idea was proposed by the research team member which comprised of academicians to the funder. This includes specifically the methodology – study design, data collection and analysis. The manuscript was written by the authors listed.<br><br>The research team regularly update the progress to the funder per requirement for monitoring of the project timeline and delivery proposed by the research team. The funder no influence in the study which would constitute conflict of interest.
2. The claim of a "virtually non-existent fire risk" is not fully substantiated by the data presented. The study only includes temperature measurements under limited conditions. Conclusions should be moderated and apply only to the scenarios analyzed.	We view that the comment is reasonable. While we maintain that the findings and results did indicate that, within the limited experimental parameters, the risk of fire is negligible, we have moderated the sentences across the manuscript to reflect academic caution.<br><br>Specifically, terms such as “no risk,” “will never be able to ignite,” and “practically absent” have been revised to confine the applicability of the findings to the limited ambient environment and conditions of the experiments conducted.<br><br>These revisions preserve the interpretation supported by the data while ensuring that the conclusions are appropriately bounded by the experimental scope.<br><br>Refer to Line 23 – 24, 38 – 40, 372 – 378, 432 – 433, and 436 – 437(Clean Copy).
3. The tests were conducted within a narrow range of environmental conditions (single location, limited temperature and humidity ranges). The influence of other factors, such as wind, extreme temperatures, and prolonged contact with the material, should be discussed.	We agree that environmental conditions such as wind, extreme temperature, humidity variation, and prolonged contact duration may influence heat transfer, material drying, smoldering behaviour, and ignition potential.<br><br>We have further expanded the Discussion and Conclusion to acknowledge that the present findings are specific to the tested products, materials, contact scenarios, and ambient laboratory conditions.

	We have also further emphasized by extending the statement that future studies should examine wider environmental conditions, including higher temperatures, lower humidity, wind exposure, aged or degraded materials, and longer contact durations.<br><br>These revisions clarify the scope of interpretation without altering the main finding that, under the tested conditions, the measured surface temperatures of HNB products remained substantially below the ignition temperatures of the evaluated combustible materials.<br><br>Refer to Line 372 – 378 and 386 - 412 (Clean Copy).
4. Please clarify the thermographic measurement parameters, particularly the emissivity settings and calibration procedure.	We thank the reviewer for highlighting the missing details on the emissivity settings and calibration of thermographic camera.<br><br>We've now included the information required which specify the calibration and the procedure to ensure quality control of the temperature measurement. This includes the emissivity values which were obtained via the black tape method and other specific details which would be expected for replicability of the study.<br><br>Refer to Line 205 – 223 (Clean Copy).
5. The description of the statistical analyses is insufficient. Please supplement with:<br>- p values and confidence intervals,<br>- justification for the number of replicates (n=3),<br>- a more detailed description of the statistical methods used.	Upon revisiting the analytical approach during revision, we recognized that the previous reference to ANOVA was not sufficiently aligned with the primary objective and design of the study. The study was intended as a descriptive thermographic assessment to characterize surface-temperature profiles and visible material effects under defined experimental conditions, rather than as an inferential statistical comparison among product types, materials, or environmental factors.<br><br>Accordingly, we have removed the ANOVA statement from the revised manuscript to avoid overinterpreting the data or implying statistical conclusions that were not central to the study objective.

	We now present the results descriptively as mean ± SD and have added extended description of the triplicate design and uncertainty/repeatability analysis. The uncertainty analysis showed that the relative variation of the recorded surface-temperature measurements was below 10%, indicating acceptable repeatability under the controlled experimental setup.<br><br>Refer to Line 160 – 167 (Clean Copy).
5. The claim that there are no previous studies may be too categorical. It's worth expanding the literature review to include related studies (e.g., those on e-cigarettes and fire safety).	We thank the reviewer for this helpful comment. We agree that the previous wording may have appeared too categorical.<br><br>Nevertheless, we would like to clarify that, prior to designing the study, we conducted a structured bibliometric review of 1041 research and review articles indexed in Scopus using predefined search terms related to heat-not-burn and heated tobacco products. Within the scope of this search, we did not identify direct experimental studies specifically evaluating the surface-temperature behaviour and contact ignition potential of HNB products under tropical ambient conditions.<br><br>Nevertheless, in response to the reviewer's comment, we have revised the manuscript to avoid implying that no related fire-safety studies exist. We now acknowledge that previous studies have examined fire risks associated with combustible cigarettes, electronic cigarettes, and other electronic nicotine delivery systems. The revised wording clarifies that the research gap addressed in this study relates specifically to direct fire-risk assessment of HNB products, particularly their external surface-temperature behaviour and contact with common combustible materials under tropical ambient conditions.<br><br>The literature review has been expanded to include studies on cigarette-related ignition of combustible materials, e-cigarette fire and explosion hazards, and thermal behaviour of electronic nicotine delivery systems.

	We now clarify that the research gap relates specifically to direct fire-risk assessment of HNB products, particularly their surface-temperature behaviour and contact with common combustible household materials under tropical ambient conditions.<br><br>Refer to Line 87 – 118 (Clean Copy).
