# Supplementary material for: Fire risk evaluation of heat-not-burn products and combustible cigarettes: Thermal exposure of different materials under ambient conditions
Source: PLoS One. 2026 Aug 7;21(8):e0351305. doi: 10.1371/journal.pone.0351305 (PMC13450766; doi:10.1371/journal.pone.0351305)
Supplement: Supplementary file 3 [file pone.0351305.s013.pdf]

UPM
UNIVERSITI PUTRA MALAYSIA
BERILMU BERBAKTI
FAKULTI PERUBATAN DAN SAINS KESIHATAN
FACULTY OF MEDICINE AND HEALTH SCIENCES

To:
Emily Chenette (Editor-in-Chief)
PLOS One (ISSN: 1932-6203)
1160 Battery Street, STE 100,
San Francisco, CA 94111
UNITED STATES OF AMERICA

Dear Editor-in-Chief,

Rebuttal Letter for PONE-D-25-38820 - Response to Original Reviewers' Comments

I hope this letter finds you well.

The Original Reviewers' Comments for the manuscript PONE-D-25-38820 as attached in the following letter were for the third part of the paper series.

The current manuscript submitted is the first part of the research was a field experiment conducted in the existing natural tropical environment in Malaysia hottest state, Perlis.

As such, this article precedes the rejected manuscript PONE-D-25-38820. I hope this clarify the situation.

Sincerely,

DR. NG YEE GUAN
Associate Professor
Universiti Putra Malaysia

NG YEE GUAN / MEDIC <shah86zam@upm.edu.my>

PLOS ONE Decision: PONE-D-25-38820 - [EMID:965dc00108c893ba]

1 message

PLOS ONE <em@editorialmanager.com> Fri, Aug 1, 2025 at 11:50 PM
Reply-To: PLOS ONE <plosone@plos.org>
To: Yee Guan Ng <shah86zam@upm.edu.my>

PONE-D-25-38820
Fire risk evaluation of heat-not-burn products and combustible cigarettes. Part 3: Flammability of fabrics and mats, and the effect of weathering
PLOS ONE

Dear Dr. Ng,

Thank you for submitting your manuscript to PLOS ONE. After careful consideration, we have decided that your manuscript does not meet our criteria for publication and must therefore be rejected.

The submission is presented as the third part of a series; however, Parts 1 and 2 are not clearly cited with publication information, nor are they accessible for evaluation. As such, the rationale and context for the current study are unclear, and we are unable to properly assess the novelty and contribution of the work.

As per the journal's guidelines on replication and derivative studies, submissions must provide a clear scientific rationale and appropriate referencing to justify the work. Unfortunately, the manuscript in its current form does not meet these requirements.

We thank you for considering PLOS ONE and encourage you to resubmit a future version that either stands independently or includes proper context and references to previously published parts.

I am sorry that we cannot be more positive on this occasion, but hope that you appreciate the reasons for this decision.

Kind regards,

Gianluca Genovese, Ph.D.
Academic Editor
PLOS ONE

In compliance with data protection regulations, you may request that we remove your personal registration details at any time. (Remove my information/details). Please contact the publication office if you have any questions.
