# Supplementary material for: Fire risk evaluation of heat-not-burn products and combustible cigarettes: Thermal exposure of different materials under ambient conditions
Source: PLoS One. 2026 Aug 7;21(8):e0351305. doi: 10.1371/journal.pone.0351305 (PMC13450766; doi:10.1371/journal.pone.0351305)
Supplement: Peer review history

# Author comment: Author response to Decision Letter 0

**DOI:** 10.1371/journal.pone.0351305.r001

17 Dec 2025

Attachment Submitted filename: Rebuttal Letter.pdf

# Peer review: Decision Letter 0

**DOI:** 10.1371/journal.pone.0351305.r002

**Authors:** Daniel Bekalo

9 Apr 2026

PONE-D-25-52903

Fire risk evaluation of heat-not-burn products and combustible cigarettes: Thermal exposure of different materials under ambient conditions

PLOS One

Dear Dr.  Ng,

Thank you for submitting your manuscript to PLOS ONE. After careful consideration, we feel that it has merit but does not fully meet PLOS ONE’s publication criteria as it currently stands. Therefore, we invite you to submit a revised version of the manuscript that addresses the points raised during the review process.

Please submit your revised manuscript by May 24 2026 11:59PM. If you will need more time than this to complete your revisions, please reply to this message or contact the journal office at plosone@plos.org. When you're ready to submit your revision, log on to https://www.editorialmanager.com/pone/ and select the 'Submissions Needing Revision' folder to locate your manuscript file.

Please include the following items when submitting your revised manuscript:

- A letter that responds to each point raised by the academic editor and reviewer(s). You should upload this letter as a separate file labeled 'Response to Reviewers'.
- A marked-up copy of your manuscript that highlights changes made to the original version. You should upload this as a separate file labeled 'Revised Manuscript with Track Changes'.
- An unmarked version of your revised paper without tracked changes. You should upload this as a separate file labeled 'Manuscript'.

If you would like to make changes to your financial disclosure, please include your updated statement in your cover letter. Guidelines for resubmitting your figure files are available below the reviewer comments at the end of this letter.

If applicable, we recommend that you deposit your laboratory protocols in protocols.io to enhance the reproducibility of your results. Protocols.io assigns your protocol its own identifier (DOI) so that it can be cited independently in the future. For instructions see: https://journals.plos.org/plosone/s/submission-guidelines#loc-laboratory-protocols. Additionally, PLOS ONE offers an option for publishing peer-reviewed Lab Protocol articles, which describe protocols hosted on protocols.io. Read more information on sharing protocols at https://plos.org/protocols?utm_medium=editorial-email&utm_source=authorletters&utm_campaign=protocols.

As the corresponding author, your ORCID iD is verified in the submission system and will appear in the published article. PLOS supports the use of ORCID, and we encourage all coauthors to register for an ORCID iD and use it as well. Please encourage your coauthors to verify their ORCID iD within the submission system before final acceptance, as unverified ORCID iDs will not appear in the published article. *Only*  the individual author can complete the verification step; PLOS staff *cannot*  verify ORCID iDs on behalf of authors.

We look forward to receiving your revised manuscript.

Kind regards,

Daniel Biftu Bekalo, PhD

Academic Editor

PLOS One

Journal Requirements:

When submitting your revision, we need you to address these additional requirements.

1.Please ensure that your manuscript meets PLOS ONE's style requirements, including those for file naming. The PLOS ONE style templates can be found at

https://journals.plos.org/plosone/s/file?id=wjVg/PLOSOne_formatting_sample_main_body.pdf and

https://journals.plos.org/plosone/s/file?id=ba62/PLOSOne_formatting_sample_title_authors_affiliations.pdf

2. We note that the grant information you provided in the ‘Funding Information’ and ‘Financial Disclosure’ sections do not match.

When you resubmit, please ensure that you provide the correct grant numbers for the awards you received for your study in the ‘Funding Information’ section.

3. Thank you for stating the following financial disclosure:

“Research funding”

Please state what role the funders took in the study.  If the funders had no role, please state: "The funders had no role in study design, data collection and analysis, decision to publish, or preparation of the manuscript."

If this statement is not correct you must amend it as needed.

Please include this amended Role of Funder statement in your cover letter; we will change the online submission form on your behalf.

4. Please provide a complete Data Availability Statement in the submission form, ensuring you include all necessary access information or a reason for why you are unable to make your data freely accessible. If your research concerns only data provided within your submission, please write "All data are in the manuscript and/or supporting information files" as your Data Availability Statement.

5. When completing the data availability statement of the submission form, you indicated that you will make your data available on acceptance. We strongly recommend all authors decide on a data sharing plan before acceptance, as the process can be lengthy and hold up publication timelines. Please note that, though access restrictions are acceptable now, your entire data will need to be made freely accessible if your manuscript is accepted for publication. This policy applies to all data except where public deposition would breach compliance with the protocol approved by your research ethics board. If you are unable to adhere to our open data policy, please kindly revise your statement to explain your reasoning and we will seek the editor's input on an exemption. Please be assured that, once you have provided your new statement, the assessment of your exemption will not hold up the peer review process.

6. Please include captions for your Supporting Information files at the end of your manuscript, and update any in-text citations to match accordingly. Please see our Supporting Information guidelines for more information: http://journals.plos.org/plosone/s/supporting-information.

7. If the reviewer comments include a recommendation to cite specific previously published works, please review and evaluate these publications to determine whether they are relevant and should be cited. There is no requirement to cite these works unless the editor has indicated otherwise.

[Note: HTML markup is below. Please do not edit.]

Reviewer's Responses to Questions

**Comments to the Author**

1. Is the manuscript technically sound, and do the data support the conclusions?

The manuscript must describe a technically sound piece of scientific research with data that supports the conclusions. Experiments must have been conducted rigorously, with appropriate controls, replication, and sample sizes. The conclusions must be drawn appropriately based on the data presented.

Reviewer #1: Yes

Reviewer #2: Partly

**********

2. Has the statistical analysis been performed appropriately and rigorously?

Reviewer #1: N/A

Reviewer #2: No

**********

3. Have the authors made all data underlying the findings in their manuscript fully available?

The PLOS Data policy requires authors to make all data underlying the findings described in their manuscript fully available without restriction, with rare exception (please refer to the Data Availability Statement in the manuscript PDF file). The data should be provided as part of the manuscript or its supporting information, or deposited to a public repository. For example, in addition to summary statistics, the data points behind means, medians and variance measures should be available. If there are restrictions on publicly sharing data—e.g. participant privacy or use of data from a third party—those must be specified.

Reviewer #1: Yes

Reviewer #2: Yes

**********

4. Is the manuscript presented in an intelligible fashion and written in standard English?

PLOS ONE does not copyedit accepted manuscripts, so the language in submitted articles must be clear, correct, and unambiguous. Any typographical or grammatical errors should be corrected at revision, so please note any specific errors here.

Reviewer #1: Yes

Reviewer #2: Yes

**********

5. Review Comments to the Author

Please use the space provided to explain your answers to the questions above. You may also include additional comments for the author, including concerns about dual publication, research ethics, or publication ethics. (Please upload your review as an attachment if it exceeds 20,000 characters)

Reviewer #1: This is a very interesting paper which presents a well-organized experimental study evaluating the fire risks associated with heated-not-burn (HNB) tobacco products. Although fire incidents are generally not expected based on the fundamental characteristics of HNB devices, the authors provide clear and objective experimental evidence demonstrating their potential as a lower (or none) fire-risk alternative to combustible cigarettes. These findings contribute useful information for assessing the broader societal acceptance of this new technology.

While discussions surrounding heated-not-burn products often focus on the chemical composition of emitted aerosols, the surface temperature profiles reported in this study offer valuable insights into the mechanisms underlying aerosol generation and the formation of associated chemical constituents. Although the study is framed in the context of use in a tropical environment such as Malaysia, the controlled experimental conditions (26°C, 67% relative humidity) support the broader relevance of the findings. Overall, the study provides meaningful academic value.

Specific comment: The authors employed a modified version of the HCI method as the puffing regime. It would be helpful if the manuscript could briefly clarify the rationale for adjusting the puff interval from 30 seconds to 60 seconds and indicate whether this modification had any measurable influence on the results.

Reviewer #2: This manuscript presents an experimental assessment of the fire risk associated with the use of heat-not-burn (HNB) products compared to traditional cigarettes, using thermographic measurements in a tropical climate. The topic is timely and relevant, and the experimental approach is generally sound. The results clearly indicate significantly lower surface temperatures of HNB devices compared to cigarettes. However, the work contains significant limitations that require consideration before publication.

1. The study was fully funded by Philip Morris International, and the conclusions clearly indicate an advantage for HNB products. Please clearly state the sponsor's role in study design, data collection, analysis, and manuscript preparation. This is crucial to maintaining transparency and demonstrating the absence of any conflict of interest.

2. The claim of a "virtually non-existent fire risk" is not fully substantiated by the data presented. The study only includes temperature measurements under limited conditions. Conclusions should be moderated and apply only to the scenarios analyzed.

3. The tests were conducted within a narrow range of environmental conditions (single location, limited temperature and humidity ranges). The influence of other factors, such as wind, extreme temperatures, and prolonged contact with the material, should be discussed. Please clarify the thermographic measurement parameters, particularly the emissivity settings and calibration procedure.

4. The description of the statistical analyses is insufficient. Please supplement with:

- p values and confidence intervals,

- justification for the number of replicates (n=3),

- a more detailed description of the statistical methods used.

5. The claim that there are no previous studies may be too categorical. It's worth expanding the literature review to include related studies (e.g., those on e-cigarettes and fire safety).

**********

6. PLOS authors have the option to publish the peer review history of their article (what does this mean?). If published, this will include your full peer review and any attached files.

If you choose “no”, your identity will remain anonymous but your review may still be made public.

**Do you want your identity to be public for this peer review?**  For information about this choice, including consent withdrawal, please see our Privacy Policy.

Reviewer #1: No

Reviewer #2: No

**********

[NOTE: If reviewer comments were submitted as an attachment file, they will be attached to this email and accessible via the submission site. Please log into your account, locate the manuscript record, and check for the action link "View Attachments". If this link does not appear, there are no attachment files.]

To ensure your figures meet our technical requirements, please review our figure guidelines: https://journals.plos.org/plosone/s/figures

You may also use PLOS’s free figure tool, NAAS, to help you prepare publication quality figures: https://journals.plos.org/plosone/s/figures#loc-tools-for-figure-preparation.

NAAS will assess whether your figures meet our technical requirements by comparing each figure against our figure specifications.

# Author comment: Author response to Decision Letter 1

**DOI:** 10.1371/journal.pone.0351305.r003

14 May 2026

Thank you for the opportunity to revise our manuscript for further consideration in PLOS ONE. We are grateful to the Academic Editor and the reviewers for their constructive comments and helpful recommendations. We have carefully revised the manuscript to address all editorial and reviewer comments.

In this revised submission, we have included the following files:

1. Response to Reviewers

2. Revised Manuscript with Track Changes

3. Clean Revised Manuscript

The manuscript has been revised to comply with PLOS ONE style requirements, including file naming and formatting. We have also reviewed and updated the funding information, financial disclosure, data availability statement, and supporting information presentation as required.

In response to the editorial requirements, we clarify the funding and role of funder statement as follows:

“The funders had no role in study design, data collection and analysis, decision to publish, or preparation of the manuscript. The funders provided vital information with regards to the HNB devices technology in order for the research team to conduct the experiments and derive the discussion.”

Please find the Financial Disclosure Statement as follow:

i) The research grant was obtained by submitting a proposal to Philip Morris Malaysia.

ii) Upon approval, there were no specific grant award number being specified by the funder.

iii) The primary research team member who received the research grant was Ng Yee Guan as the project leader who manages the research fund under the Research Management Centre of the University.

iv) All research team members are students and/or employees of Universities as provided in their affiliation during the research project.

v) The website URL of the funder is as follow: https://www.pmi.com/markets/malaysia/en/about-us/overview

Following the integration of the Supplementary Information into the main manuscript, we have also revised the Data Availability Statement to state:

“All data are in the manuscript file.”

Substantive revisions have been made in response to reviewer comments. These include clarification of the hybrid/modified puffing regime, specifically the use of the Health Canada Intense puff volume and duration with the ISO 3308 puff interval. We have also clarified the rationale for using a 60 s puff interval and acknowledged that the direct influence of this modification was not separately quantified because a parallel 30 s comparison was not included.

We have moderated categorical claims such as “no risk,” “will never be able to ignite,” and “practically absent” to ensure that the conclusions are appropriately bounded by the tested products, materials, and experimental conditions. The Discussion and Conclusion have also been expanded to address limitations related to the narrow range of environmental conditions, including the potential influence of wind, extreme temperature, humidity variation, prolonged contact duration, and material condition.

In addition, we have clarified the thermographic measurement parameters, including emissivity settings and calibration procedures. The statistical description has also been revised. The previous ANOVA statement was removed to avoid overinterpretation, and the study is now presented as a descriptive thermographic assessment supported by triplicate measurements and uncertainty/repeatability analysis.

We have also expanded the literature review to include related studies on combustible cigarettes, e-cigarette fire safety, and thermal behaviour of electronic nicotine delivery systems, while clarifying that the specific gap addressed by this study concerns HNB surface-temperature behaviour and contact fire-risk potential under tropical ambient conditions.

We believe that these revisions have strengthened the manuscript, improved methodological transparency, and ensured that the conclusions are appropriately supported by the data. We appreciate the time and effort of the Academic Editor and reviewers in evaluating our work.

Thank you for considering our revised manuscript.

Attachment Submitted filename: Letter - Response to Editor.pdf

# Peer review: Decision Letter 1

**DOI:** 10.1371/journal.pone.0351305.r004

**Authors:** David Greenhalgh

27 May 2026

Fire risk evaluation of heat-not-burn products and combustible cigarettes: Thermal exposure of different materials under ambient conditions

PONE-D-25-52903R1

Dear Dr. Ng,

We’re pleased to inform you that your manuscript has been judged scientifically suitable for publication and will be formally accepted for publication once it meets all outstanding technical requirements.

Within one week, you’ll receive an e-mail detailing the required amendments. When these have been addressed, you’ll receive a formal acceptance letter and your manuscript will be scheduled for publication.

An invoice will be generated when your article is formally accepted. Please note, if your institution has a publishing partnership with PLOS and your article meets the relevant criteria, all or part of your publication costs will be covered. Please make sure your user information is up-to-date by logging into Editorial Manager at Editorial Manager® and clicking the ‘Update My Information' link at the top of the page. For questions related to billing, please contact billing support.

If your institution or institutions have a press office, please notify them about your upcoming paper to help maximize its impact. If they’ll be preparing press materials, please inform our press team as soon as possible -- no later than 48 hours after receiving the formal acceptance. Your manuscript will remain under strict press embargo until 2 pm Eastern Time on the date of publication. For more information, please contact onepress@plos.org.

Kind regards,

David G. Greenhalgh, MD

Academic Editor

PLOS One

Additional Editor Comments (optional):

Accept

Reviewers' comments:

Reviewer's Responses to Questions

**Comments to the Author**

1. If the authors have adequately addressed your comments raised in a previous round of review and you feel that this manuscript is now acceptable for publication, you may indicate that here to bypass the “Comments to the Author” section, enter your conflict of interest statement in the “Confidential to Editor” section, and submit your "Accept" recommendation.

Reviewer #1: All comments have been addressed

Reviewer #2: All comments have been addressed

**********

2. Is the manuscript technically sound, and do the data support the conclusions?

The manuscript must describe a technically sound piece of scientific research with data that supports the conclusions. Experiments must have been conducted rigorously, with appropriate controls, replication, and sample sizes. The conclusions must be drawn appropriately based on the data presented.

Reviewer #1: Yes

Reviewer #2: Yes

**********

3. Has the statistical analysis been performed appropriately and rigorously?

Reviewer #1: Yes

Reviewer #2: Yes

**********

4. Have the authors made all data underlying the findings in their manuscript fully available?

The PLOS Data policy requires authors to make all data underlying the findings described in their manuscript fully available without restriction, with rare exception (please refer to the Data Availability Statement in the manuscript PDF file). The data should be provided as part of the manuscript or its supporting information, or deposited to a public repository. For example, in addition to summary statistics, the data points behind means, medians and variance measures should be available. If there are restrictions on publicly sharing data—e.g. participant privacy or use of data from a third party—those must be specified.

Reviewer #1: Yes

Reviewer #2: Yes

**********

5. Is the manuscript presented in an intelligible fashion and written in standard English?

PLOS ONE does not copyedit accepted manuscripts, so the language in submitted articles must be clear, correct, and unambiguous. Any typographical or grammatical errors should be corrected at revision, so please note any specific errors here.

Reviewer #1: Yes

Reviewer #2: Yes

**********

6. Review Comments to the Author

Please use the space provided to explain your answers to the questions above. You may also include additional comments for the author, including concerns about dual publication, research ethics, or publication ethics. (Please upload your review as an attachment if it exceeds 20,000 characters)

Reviewer #1: (No Response)

Reviewer #2: The subject of the manuscript focused on fire risk evaluation of heat-not-burn products and combustible cigarettes and thermal exposure of different materials under ambient conditions is in good relevance with the scope of PLOS ONE. The topic is timely and relevant, and the experimental approach is generally sound. The results clearly indicate significantly lower surface temperatures of HNB devices compared to cigarettes.

The authors responded in detail to all comments included in the review and made necessary methodological changes and additions to the manuscript. In its current form, the manuscript is suitable for publication after making the necessary editorial and other corrections.

**********

7. PLOS authors have the option to publish the peer review history of their article (what does this mean?). If published, this will include your full peer review and any attached files.

If you choose “no”, your identity will remain anonymous but your review may still be made public.

**Do you want your identity to be public for this peer review?**  For information about this choice, including consent withdrawal, please see our Privacy Policy.

Reviewer #1: No

Reviewer #2: No

**********

# Editor report: Acceptance letter

**DOI:** 10.1371/journal.pone.0351305.r005

**Authors:** David Greenhalgh

PONE-D-25-52903R1

PLOS One

Dear Dr. Ng,

I'm pleased to inform you that your manuscript has been deemed suitable for publication in PLOS One. Congratulations! Your manuscript is now being handed over to our production team.

At this stage, our production department will prepare your paper for publication. This includes ensuring the following:

* All references, tables, and figures are properly cited

* All relevant supporting information is included in the manuscript submission,

* There are no issues that prevent the paper from being properly typeset

You will receive further instructions from the production team, including instructions on how to review your proof when it is ready. Please keep in mind that we are working through a large volume of accepted articles, so please give us a few days to review your paper and let you know the next and final steps.

Lastly, if your institution or institutions have a press office, please let them know about your upcoming paper now to help maximize its impact. If they'll be preparing press materials, please inform our press team within the next 48 hours. Your manuscript will remain under strict press embargo until 2 pm Eastern Time on the date of publication. For more information, please contact onepress@plos.org.

You will receive an invoice from PLOS for your publication fee after your manuscript has reached the completed accept phase. If you receive an email requesting payment before acceptance or for any other service, this may be a phishing scheme. Learn how to identify phishing emails and protect your accounts at https://explore.plos.org/phishing.

If we can help with anything else, please email us at customercare@plos.org.

Thank you for submitting your work to PLOS ONE and supporting open access.

Kind regards,

PLOS ONE Editorial Office Staff

on behalf of

Dr. David G. Greenhalgh

Academic Editor

PLOS One
